# Post-traumatic splenic cysts treated with laparoscopy: two case reports

**DOI:** 10.4076/1757-1626-2-7976

**Published:** 2009-07-24

**Authors:** Dimitrios V Avgerinos, Christos E Kyriakopoulos, Sophia Konstantinopoulou, Evgenia Gourgari, Vasileios Lioutas

**Affiliations:** 1 Department of Surgery, Beth Israel Medical Center, Albert Einstein College of MedicineFirst Avenue at 16^th^ Street, New YorkUSA; 2 Department of Medicine, Beth Israel Medical Center, Albert Einstein College of MedicineNew YorkUSA; 3 Department of Pediatrics, Monmouth Medical Center300 Second Avenue, Long Branch, New JerseyUSA; 4 Department of Pediatrics, Miami Children’s Hospital3100 SW 62^nd^ Avenue, Miami, FloridaUSA; 5 Department of Medicine, Montefiore Medical Center, Albert Einstein College of Medicine111 East 210^th^ Street, Bronx, New YorkUSA; 6 Athens Medical InternationalLykavittou 6, Kolonaki, AthensGreece

## Abstract

**Introduction:**

Today, laparoscopy tends to become a useful alternative to open procedure for the surgical treatment of spleen disorders, offering at the same time a conservative approach for the management of selected spleen lesions such as posttraumatic cysts.

**Case presentation:**

This article describes two cases of posttraumatic splenic cysts, one of which was treated with laparoscopic total splenectomy and the second with laparoscopic cystectomy. The procedure was carried out successfully with no complications in both cases, and the patients were discharged a few days after the operation.

**Conclusion:**

Laparoscopy with preservation of functional splenic parenchyma, when feasible, should be the procedure of choice in cases of posttraumatic splenic cysts, as it provides safe and definite treatment with all of the other advantages of minimally invasive surgery.

## Introduction

The great success and acceptance of laparoscopic cholecystectomy has developed strong trends towards the modification of nearly all intra-abdominal operations to laparoscopically-assisted procedures. Nevertheless, not all of these attempts achieved the same amount of success [1]. Regarding the surgery of the spleen, laparoscopy substituted the traditional open procedure in most cases, and nowadays the vast majority of surgical centers regard laparoscopic splenectomy as the procedure of choice for patients requiring routine splenectomy [2].

In the case of splenic cysts, the first trend suggested total splenectomy as the procedure of choice [3]. However, following pathophysiologic considerations, surgeons started using conservative methods, in an attempt to reduce the risk of postsplenectomy pneumococcal infection and preserve the long-term immunological role of the spleen [4]. As a result, today laparoscopy has been employed in the treatment of all kinds of splenic cysts, either by total splenectomy or by conservative techniques, when feasible. We report on two female patients who received laparoscopic treatment of posttraumatic cyst.

## Case presentations

### Case report 1

**Figure 1.**
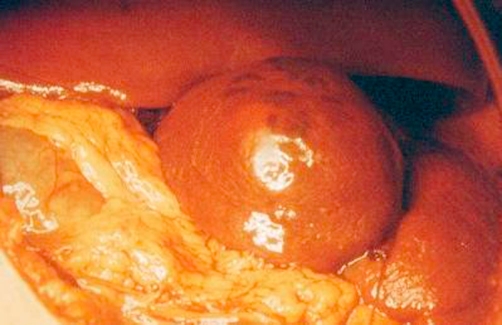
Intra-operative photo, showing the splenic cyst.

A 38-year-old Caucasian woman presented to our clinic with the major complaint of abdominal fullness and left upper quadrant heaviness. The history mentioned a blunt abdominal trauma three years earlier. On physical examination, a large painless tumor was found in the left upper quadrant. The tumor was not mobile with the turning of the patient. Hematology was indiscriminatory, whereas tumor markers and indications for parasites were negative. Ultrasound revealed a large cyst evolving from the spleen and computed tomography (CT) showed a 5 × 4 cm cystic lesion of the spleen. The cyst was resected along with the spleen, using a standard technique laparoscopic splenectomy (Figure 1). Pathologic examination displayed a fibrotic cystic wall with no epithelial lining, indicating a posttraumatic pseudocyst of the spleen. Postoperative recovery was regular and painless.

### Case report 2

**Figure 2.**
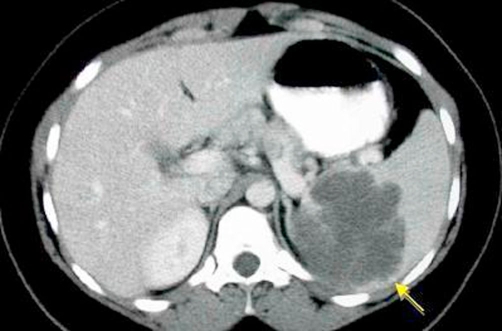
CT scan of the splenic cyst (arrow).

A 37-year-old Caucasian woman presented to our hospital with acute abdominal pain after trauma in the left lower hemithorax seven hours prior. Computed tomography (CT) revealed a 5 cm cyst with liquid content, occupying the upper pole of the spleen, but no involvement of the liver, kidney and chest (Figure 2). Laparoscopic exploration and excision of the lesion was decided. A standard laparoscopic cystectomy was performed with preservation of the remaining spleen. The results of the histological examination were: “Splenic cyst with 28 g of weight and dimensions of 5 × 4 × 2 cm, containing 17 cm^3^ of serous-bloody liquid. The cystic wall is fibrotic with epithelial lining, indicating a posttraumatic splenic pseudocyst”. No postoperative complications occurred and the patient was discharged five days after surgery with a negative abdominal ultrasound examination.

## Discussion

After the overwhelming success of laparoscopic cholecystectomy, laparoscopic techniques have been applied to various surgical conditions of the abdomen, in an attempt to achieve analogous improvements [5]. The spleen could not be an exception to this trend. First introduced in 1991 [6,7], laparoscopic splenectomy has undergone significant refinement as surgeons’ experience has improved and as new technology has been developed.

Laparoscopy has also been employed in the management of splenic cysts of all kinds. All types of splenic cysts are rare [3]. In a review of 42,327 autopsies, splenic cysts were found to have an incidence of 0.07% [8]. All the same, even after trauma, posttraumatic splenic cysts are rarely seen [9]. Tsakayannis *et al* reported on two cases of splenic cysts with a history of preceding trauma out of 19 patients [10], and Touloukian *et al* found only one out of six such cases [11]. Splenic cysts are classified as “true” and “pseudo” cysts, based on the presence or absence of an epithelial lining, respectively [10]. True cysts are subdivided into parasitic and nonparasitic, whereas pseudocysts are believed to be a late manifestation of posttraumatic intrasplenic hematoma [10] and represent 75% of all splenic cysts [12].

Before the 1980s, the treatment of choice for cysts of the spleen was total splenectomy [3]. Nevertheless, the importance of preserving the spleen, especially in children, has been well recognized [13]. As a result, the understanding of the short- and long-term complications of splenectomy, such as postsplenectomy sepsis, has led to the practice of splenic conservation when feasible [10,14]. It is believed that preservation of at least 25% of the spleen offers protection against pneumococcal bacteremia [15]. The most commonly used conservative procedures are partial splenectomy and total cystectomy with splenorrhaphy [10,16,17]. In many cases of splenic pseudocysts, partial splenic decapsulation (partial cyst excision and marsupialization) is also used [9]. Feliciotti *et al* reported on two patients with posttraumatic splenic cyst who were treated with laparoscopic total cystectomy and splenorrhaphy, using a specifically developed intra-abdominal ultrasound probe in order to accurately determine the thinnest splenic parenchyma bordering the cyst [4]. Percutaneous drainage of the cyst under ultrasound or CT guidance is a safe procedure, but has a high recurrence risk. In posttraumatic cysts the fluid tends to reaccumulate probably because of osmotically active debris and destruction of normal splenic architecture [18].

The clinical manifestation of the splenic cysts varies accordingto their size, location and complications. Most patients present with minor, non-specific symptoms of local compression of adjacent structures, such as local or referred pain and abdominal distension [18]. Splenic cysts may also be discovered after becoming infected, ruptured or hemorrhagic [19,20]. Besides, many cysts are found accidentally during physical or radiologic exam [19]. All cysts of the spleen that are symptomatic or larger than 5 cm should receive surgical treatment, as they are in high risk of being ruptured [19].

## Conclusion

Laparoscopy offers satisfactory visualization of the spleen. The avoidance of an upper abdominal incision leads to low postoperative pain and discomfort of the patient as well as to short length of hospital stay. It is a surgical procedure that offers complete treatment, better quality of life and increased self-manageability of the patient during the postoperative period, lower postoperative morbidity and mortality and, of course, better cosmetic results, which is essential to female and especially to younger patients, who are prone to traumatic lesions of the spleen. As a result, we believe that laparoscopy with preservation of functional splenic parenchyma, when feasible, should be the procedure of choice in patients with posttraumatic cysts of the spleen, as it provides safe and definite treatment with all the other advantages of laparoscopic surgery.

## Abbreviation

CT: computed tomography
